# NMR Metabolomics Reveal Urine Markers of Microbiome Diversity and Identify Benzoate Metabolism as a Mediator between High Microbial Alpha Diversity and Metabolic Health

**DOI:** 10.3390/metabo12040308

**Published:** 2022-03-31

**Authors:** Johannes Hertel, Daniel Fässler, Almut Heinken, Frank U. Weiß, Malte Rühlemann, Corinna Bang, Andre Franke, Kathrin Budde, Ann-Kristin Henning, Astrid Petersmann, Uwe Völker, Henry Völzke, Ines Thiele, Hans-Jörgen Grabe, Markus M. Lerch, Matthias Nauck, Nele Friedrich, Fabian Frost

**Affiliations:** 1 Department of Psychiatry and Psychotherapy, University Medicine Greifswald, D-17475 Greifswald, Germany; daniel.faessler@uni-greifswald.de (D.F.); hans.grabe@med.uni-greifswald.de (H.-J.G.); 2 School of Medicine, National University of Ireland, H91 CF50 Galway, Ireland; almut-katrin.heinken@univ-lorraine.fr (A.H.); ines.thiele@nuigalway.ie (I.T.); 3 Department of Internal Medicine A, University Medicine Greifswald, D-17475 Greifswald, Germany; ulrich.weiss@med.uni-greifswald.de (F.U.W.); markus.lerch@med.uni-muenchen.de (M.M.L.); fabian.frost@med.uni-greifswald.de (F.F.); 4 Institute of Clinical Molecular Biology, Kiel University, D-24105 Kiel, Germany; m.ruehlemann@ikmb.uni-kiel.de (M.R.); c.bang@ikmb.uni-kiel.de (C.B.); a.franke@mucosa.de (A.F.); 5 Institute of Clinical Chemistry and Laboratory Medicine, University Medicine Greifswald, D-17475 Greifswald, Germany; kathrin.budde@med.uni-greifswald.de (K.B.); ann-kristin.henning@med.uni-greifswald.de (A.-K.H.); petersmann.astrid@klinikum-oldenburg.de (A.P.); matthias.nauck@med.uni-greifswald.de (M.N.); nele.friedrich@med.uni-greifswald.de (N.F.); 6 Institute of Clinical Chemistry and Laboratory Medicine, University Medicine Oldenburg, D-26129 Oldenburg, Germany; 7 Department of Functional Genomics, Interfaculty Institute for Genetics and Functional Genomics, University Medicine Greifswald, D-17475 Greifswald, Germany; voelker@uni-greifswald.de; 8 Institute for Community Medicine, University of Greifswald, D-17475 Greifswald, Germany; voelzke@uni-greifswald.de; 9 Discipline of Microbiology, National University of Galway, H91 CF50 Galway, Ireland; 10 APC Microbiome Ireland, University College Cork, T12 CY82 Cork, Ireland; 11 Ryan Institute, National University of Galway, H91 CF50 Galway, Ireland; 12 German Center for Neurodegenerative Diseases (DZNE), Partner Site Rostock/Greifswald, D-17475 Greifswald, Germany; 13 Faculty of Medicine, Ludwig-Maximilian University Munich, D-80539 Munich, Germany; 14 German Centre for Cardiovascular Research (DZHK), Partner Site, D-17475 Greifswald, Germany

**Keywords:** NMR metabolomics, microbiome, large cohort data, alpha diversity, benzoate metabolism

## Abstract

Microbial metabolites measured using NMR may serve as markers for physiological or pathological host–microbe interactions and possibly mediate the beneficial effects of microbiome diversity. Yet, comprehensive analyses of gut microbiome data and the urine NMR metabolome from large general population cohorts are missing. Here, we report the associations between gut microbiota abundances or metrics of alpha diversity, quantified from stool samples using 16S rRNA gene sequencing, with targeted urine NMR metabolites measures from 951 participants of the Study of Health in Pomerania (SHIP). We detected significant genus–metabolite associations for hippurate, succinate, indoxyl sulfate, and formate. Moreover, while replicating the previously reported association between hippurate and measures of alpha diversity, we identified formate and 4-hydroxyphenylacetate as novel markers of gut microbiome alpha diversity. Next, we predicted the urinary concentrations of each metabolite using genus abundances via an elastic net regression methodology. We found profound associations of the microbiome-based hippurate prediction score with markers of liver injury, inflammation, and metabolic health. Moreover, the microbiome-based prediction score for hippurate completely mediated the clinical association pattern of microbial diversity, hinting at a role of benzoate metabolism underlying the positive associations between high alpha diversity and healthy states. In conclusion, large-scale NMR urine metabolomics delivered novel insights into metabolic host–microbiome interactions, identifying pathways of benzoate metabolism as relevant candidates mediating the beneficial health effects of high microbial alpha diversity.

## 1. Introduction

An incredible wealth of research has highlighted the importance of the gut microbiome’s metabolic activity to health and disease [1,2,3,4,5]. Microbial metabolites, such as short-chain fatty acids or vitamin derivatives, are beneficial for health [6,7,8], while others, such as hydrogen sulphide [9,10], secondary bile acids [11,12,13], or trimethylamine-N-oxide [14,15], are involved in pathophysiological processes. Yet, our understanding of the complex interplay between the microbiome and host metabolism is incomplete, in particular because there is no bijection between gut microbiome composition and its metabolic activity. As metabolic functions widely overlap across the main bacterial phyla in the gut, two microbial communities may diverge strongly in their composition but be largely equivalent in their metabolic functions [16]. Moreover, since the gut microbiome acts within the unique ecosystem shaped by host factors, such as behaviour [17], genetics [18,19,20,21], and physiology [22,23], two gut microbiomes that are largely equivalent in metabolic function may lead to different health outcomes. Thus, the question arises regarding how alterations in composition translate into differences in metabolic host functions.

The mechanisms conferring the health-promoting effects of high alpha diversity are of particular interest [24]. Multiple studies have revealed associations of reduced alpha diversity with a wide range of disease phenotypes, including gastrointestinal disorders [25,26], obesity [27], or diabetes [28,29,30,31,32]. While the hypothesis exists that high diversity implicates a higher functional redundancy and thus more stable host–microbiome interactions [33], the concrete metabolic traits underlying the relation between microbial diversity and human health remain elusive.

A frequently utilised paradigm to shine a light on the complex interplay between gut microbiome composition and metabolic function in the host is the ‘ome-wide’ association study. In this paradigm, the statistical association pattern between abundances of taxonomic units and metabolite concentrations in the host is derived via sequential multivariable regressions. ‘Ome-wide’ association studies can serve as powerful tools for discovery and hypotheses generation. For example, we previously showed that reduced microbial diversity is associated with long-term microbiome instability, which is a phenotype characterised by an increase in the microbial biosynthesis capability for proinflammatory lipopolysaccharides over time [33]. Moreover, Wilmanski et al. [34] found that blood metabolites were highly predictive of the gut microbiome alpha diversity with prominent roles for human–microbial co-metabolites, such as hippurate, cinnamoylglycine, and p-cresol sulfate. However, microbiome–metabolome association studies on large cohorts are still scarce such that putative associations are often in need of validation.

Following these lines of research, the overarching goal of this study was to broaden the understanding of metabolite–microbiome association patterns by using data from the TREND cohort of the Study of Health in Pomerania [35] (SHIP), which is a population-based cohort study from North-Eastern Germany, where subsets were characterised by targeted ^1^H nuclear magnetic resonance (NMR) urine metabolomics and faecal 16S rRNA gene sequencing. Importantly, rich phenotype data for the SHIP-TREND participants were also available, allowing for comprehensive covariate adjustments. NMR urine metabolomics is especially valuable in the context of microbiome research since a wide range of topical microbiome-related metabolites, such as indoxyl-3-sulphate, trimethylamine-N-oxide, formate, or acetate, can be readily quantified with high accuracy in the human urine through NMR technology. To the best of our knowledge, no large cohort study has been published using NMR urine metabolomics and 16S rRNA gene-sequencing data in conjunction to explore association patterns between the microbiome and host metabolome.

We herein report (1) the urine metabolite–genus association pattern, (2) the urine metabolome association pattern with measures of microbiome alpha diversity, (3) the results of machine learning modelling predicting the urine metabolites from microbiome abundances, and (4) the clinical association pattern of the microbiome-based hippurate prediction score in relation to the Shannon diversity. Finally, we explore the relationship between microbial benzoate metabolism and alpha diversity via functional annotations of an independent metagenomics dataset. In conjunction, our results highlight the usefulness of NMR metabolomics in exploring host–microbiome association patterns, while indicating that benzoate metabolism is one of the mediating metabolic functions underlying the association between alpha diversity and human health.

## 2. Results

In SHIP-TREND, gut microbiome quantifications from stool samples using 16S sequencing were available for 3637 individuals, from which, 950 were additionally characterised using NMR urine metabolomics. The sample characteristics are given in Table 1. As known diabetes cases were excluded from NMR characterisation for reasons unrelated to the presented study, diabetes-related comorbidities and physiological traits were under-represented in the sample with NMR characterisation. Thus, the NMR analyses were performed on a predominantly healthy population. For more information on sample selection, see the Methods section.

### 2.1. NMR Metabolomics Revealed Genus Metabolite Associations

The utilised panel of metabolite quantifications contained 60 metabolites, from which, 42 were included in analyses after excluding all metabolites with more than 50% missing quantifications. All metabolite concentrations were log-transformed after regression-based normalisation [36], and outliers, as defined using the 4-SD rule, were excluded from analyses. The final panel of metabolites included a wide range of metabolites known to be implicated in host–microbiome interactions. Descriptive statistics for all metabolites measured can be found in Supplementary Table S1.

The metabolite concentrations were regressed in multivariable mixed linear regressions on the genus abundances, including known influence factors of the urine metabolome and the microbiome as covariates to reduce residual confounding and using batch as the random effects variable (see the Methods section for details). We ran two sets of metabolite genus associations: (1) we included the genus presence (binary: present vs. absent) as the predictor of interest, (2) we utilised the genus abundance (dimensional, %) as the predictor of interest. Those two sets of analyses are not equivalent, as it is known that sometimes the presence of a certain species or genus may be more informative than its abundance, especially in the case of keystone species contributing important metabolic capabilities to the community [37,38].

**Figure 1:**
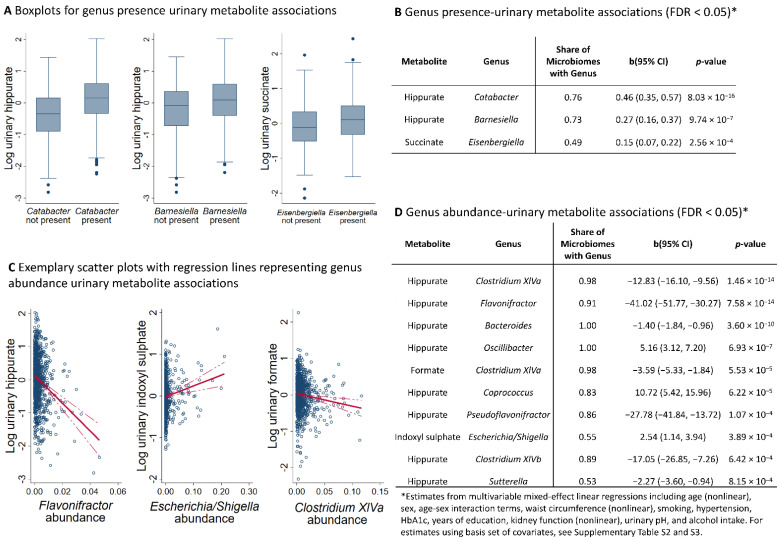
**Overview of the significant metabolite–genus associations.** (**A**) Boxplots for genus presence metabolite association with a false discovery rate < 0.05. The Y-axis denotes the log-transformed and regression-normalised urinary concentrations. (**B**) Table displaying the genus presence metabolite associations with FDR < 0.05. (**C**) Scatter plots of selected genus abundances against urinary metabolite levels after log-transformation and regression-based normalisation. The red line indicates the linear regression line, where the dashed red lines display the 95% confidence interval. (**D**) Table displaying the genus abundance metabolite associations with FDR < 0.05. FDR—false discovery rate; b—unstandardised regression coefficients; 95% CI—95% confidence intervals.

After correction for multiple testing, we found three genus presence metabolite associations (Figure 1A,B), with urinary hippurate being positively associated with the presence of *Catabacter* and *Barnesialla* and urinary succinate being positively associated with *Eisenbergiella*. In the domain of genus abundances, we detected ten associations after correction for multiple testing (Figure 1C,D). Hippurate was associated with nine different genera, while formate was additionally negatively associated with the *Clostridium XIVa* cluster and indoxyl sulfate was positively associated with *Escherichia/Shigella*. Both sets of associations remained stable upon full covariate adjustments (Figure 1B,D). In the sensitivity analyses, by utilising non-parametric bootstrapping for *p*-value calculation, all abundance metabolite associations stayed significant. However, the *p*-value of the indoxyl sulfate *Escherichia/Shigella* association dropped to nominal significance, indicating that this specific result may need further validation. The summary statistics of the corresponding association analyses can be found in Supplementary Tables S2 and S3.

### 2.2. NMR Metabolomics Revealed Markers of Microbial Alpha Diversity

Next, we screened the urine metabolome for markers of alpha diversity to investigate species richness and the Shannon entropy following the same regression methodology as above.

**Figure 2:**
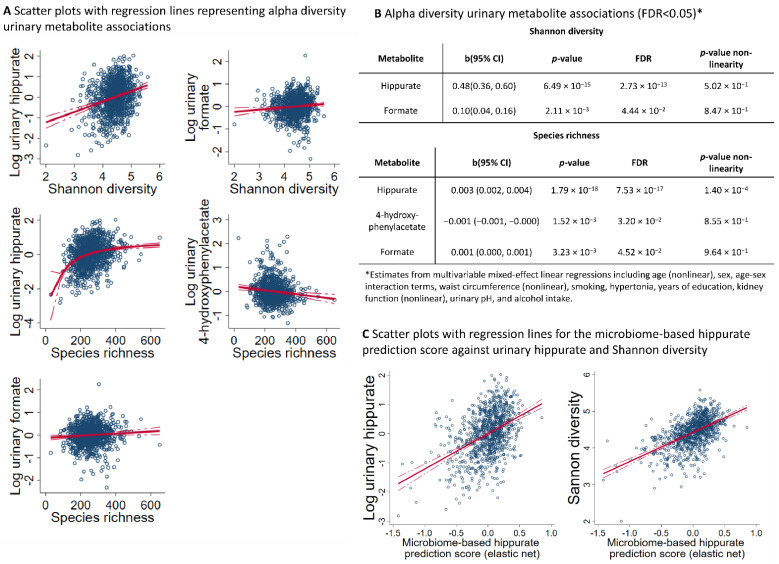
**Overview of microbiome alpha diversity–metabolite associations**. (**A**) Scatter plots of Shannon diversity or species richness against urinary metabolite levels after log-transformation and regression-based normalisation. A red line indicates the linear regression line, where the dashed red lines display the 95% confidence interval. All shown associations have an FDR < 0.05. (**B**) Table displaying the alpha diversity metabolite associations with an FDR < 0.05. (**C**) Scatter plots of Shannon diversity or urinary hippurate concentrations (log-transformed and regression-based normalised) against the microbiome-based hippurate prediction score from elastic net regressions. A red line indicates the linear regression line, where the dashed red lines display the 95% confidence interval. FDR—false discovery rate; b—unstandardised regression coefficients; 95% CI—95% confidence intervals.

After correction for multiple testing, we retrieved two associations regarding Shannon entropy and three associations regarding species richness. The top hit of the analyses was hippurate (Figure 2A,B). Additionally, we found two additional markers of microbiome diversity: formate was positively associated with both species richness and Shannon entropy, while 4-hydroxyphenylacetate was negatively associated with species richness. Complete results on metabolite diversity associations can be found in Supplementary Tables S4 and S5.

### 2.3. Microbiome-Based Predictions Scores for Urinary Hippurate Mediated the Associations of SHANNON Diversity to Markers of Metabolic Health

Then, we generated prediction scores for each metabolite using the genus abundance data as input for elastic net regressions (see the Methods section for details), assessing the model validity via internal cross-validations. However, the machine learning approach only led to substantial R-squared values for hippurate (Figure 2C), reaching 12.7% of the explained variance, whereas, for all other metabolites, the explained variance was below 3%, as determined in cross-validations (Supplementary Table S6). Therefore, we focused on the microbiome-based hippurate prediction score in further analyses.

Interestingly, the microbiome-based hippurate prediction score was substantially correlated (r = 0.53; 95% CI: 0.49, 0.58; *p* = 1.07 × 10^−71^) with measures of alpha diversity (Figure 2C). Furthermore, in fully adjusted models, the microbiome-based hippurate prediction score showed robust negative associations with markers of human diseases (Table 2), including inflammation markers (high-sensitive C-reactive protein), triglyceride levels, and, most prominently, markers of liver injury (plasma concentrations of gamma-glutamyl transferase (GGT), alanine aminotransferase (ALAT), and aspartateaminotransferase (ASAT)). Notably, the association pattern was more pronounced than for both the Shannon entropy and urinary hippurate levels themselves.

Given the strong association of the hippurate prediction score with the Shannon entropy, we tested whether the hippurate prediction score could mediate the associations of the Shannon entropy with liver markers (GGT and ALAT) and triglycerides. Indeed, in all three cases, the microbiome-based hippurate prediction score mediated the associations nearly completely (GGT: 77.6% of the effect mediated, 95% CI: 56.8%, 119.7%; ALAT: 116.7% of the effect mediated, 95% CI: 64.6%, 432.1%; triglycerides: 77.9% of the effect mediated, 95% CI: 54.3%, 132.5%). Note that mediation effects of more than 100% indicate that the direct effect had a different sign than the total effect.

Thus, the microbiome-based prediction score for hippurate contained substantial information on markers of human health and mediated the corresponding effects of microbial alpha diversity.

### 2.4. Functional Annotations of an Independent Published Dataset Indicated a Direct Relationship between Microbial Diversity and Benzoate Metabolism

Above, we show the results of the analysed association patterns between urinary metabolites and microbiome diversity via statistical screening approaches. To strengthen our findings, we used an independent public dataset with metagenomic gut microbiome characterisations from Yachida et al. (*n* = 616) [39] (see Table S7 for sample characteristics) to analyse the relationship between microbial benzoate metabolism and microbial diversity. Note that the study of Yachida et al. [39] was a case-control study investigating colorectal cancer (CRC).

**Figure 3:**
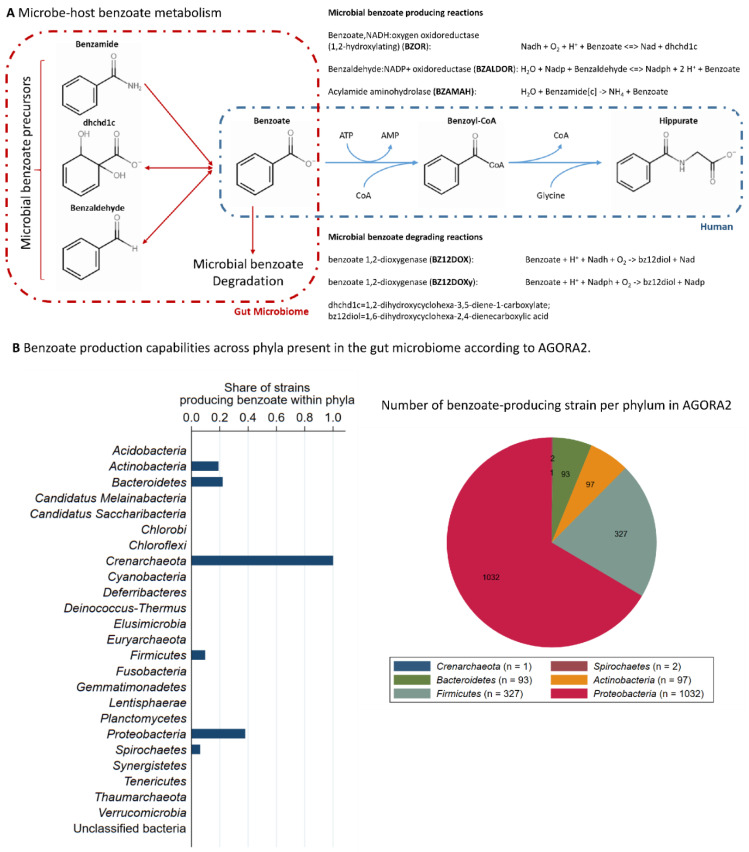
**Microbe–host interactions regarding benzoate metabolism.** (**A**) Microbe–host benzoate co-metabolism, as noted in AGORA2 and the Virtual Metabolic Human database (https://www.vmh.life, accessed on 28 January 2022). The reaction naming follows the AGORA2 nomenclature. (**B**) Benzoate production capabilities across phyla, as noted in AGORA2 as a share within phyla (left panel) and absolute number (right panel).

For functional annotations, we mapped the species abundances as reported in the supplement of Yachida et al. [39] onto AGORA2 [40]. AGORA2 is a resource of over 7000 genome-scale reconstructions of gut microbes that were semi-automatically generated and manually curated to match the experimental behaviour of the microbes. We then calculated reaction abundances for all microbial reactions that either produced or degraded benzoate (Figure 3A). Benzoate is the precursor of the human metabolite hippurate, which is predominantly produced in the liver through glycine conjugation (Figure 3A). Note that reaction abundances are not equivalent to gene abundances, as several genes may facilitate the same reactions and one gene may catalyse different reactions.

**Figure 4:**
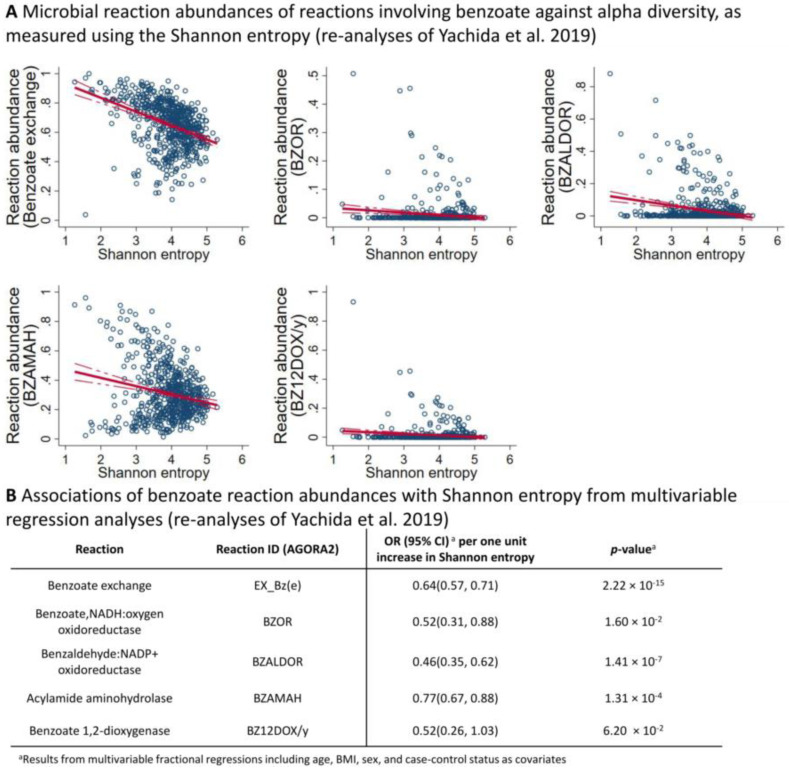
**Overview of the results regarding reaction abundances after functional annotation of the Yachida et al. dataset using AGORA2.** (**A**) Scatter plots of reaction abundances against the Shannon entropy of metagenomes after mapping onto AGORA2. A red line indicates the linear regression line, where the dashed red lines display the 95% confidence interval. All displayed associations had a false discovery rate < 0.05. (**B**) Table displaying the reaction abundance Shannon entropy associations for benzoate-producing, -degrading, or -transporting reactions noted in AGORA2. OR—odds ratio; 95% CI—95% confidence interval.

Then, we calculated the association between microbial diversity measured through the Shannon entropy and the reaction abundances of reactions either degrading, producing, or transporting benzoate (Figure 4A,B). Surprisingly, in all cases, we found a negative association between reaction abundances and Shannon entropy, which was most pronounced for the benzoate exchange reaction. In the sensitivity analyses, we tested whether these findings held up in both study groups of the Yachida et al. study (healthy controls and CRC patients), finding that this is a robust result regarding health and at least one disease known to alter the gut microbiome profoundly.

Furthermore, we utilised AGORA2 to retrieve the three biomarkers (benzoate, formate, and 4-hydroxyphenylacetate) of microbial diversity from the list of strains included in AGORA2 with the capacity to produce them (Supplementary Table S8). The results per phyla for benzoate are given in Figure 3B. Notably, benzoate production capabilities are distributed across several of the main phyla in the gut, including Actinobacteria, Bacteroidetes, Proteobacteria, and Firmicutes.

In conclusion, our functional analyses of the published data from Yachida et al. indicated a direct relationship between microbial diversity and benzoate metabolisation. However, the indicated relationship was negative with highly diverse communities showing lower reaction abundances of reactions transporting, degrading, or producing benzoate.

## 3. Discussion

Although immense research efforts have been undertaken, we are still at the beginning of understanding the multitude of effects the microbiome has on human health and disease. One key to understanding the relationship between the host and microbiome is to unravel the metabolic interplay between human metabolism and microbiome metabolism. This study contributed to the latter by investigating the urine metabolome via NMR technology [41] in conjunction with 16S rRNA gene sequencing of stool samples stemming from a large population-based study.

To date, a wide range of studies associating the urine metabolome with the gut microbiome have been performed, revealing a rich association pattern between the two types of omics data in various setups [42,43,44,45,46,47,48]. However, only a few studies have so far utilised NMR technology, and those studies were small studies relying on small-to-medium sample sizes [49,50,51,52]. Our present work was, to the best of our knowledge, the largest study so far that made use of NMR metabolomics to reveal the association patterns between the urine metabolome and the gut microbiome.

As NMR technology allows for quantifications of a wide range of microbiome-related metabolites, such as dimethylamine, formate, methanol, and acetate, which are normally not included in mass-spectrometric analyses, we could deliver novel insights while replicating earlier results, in particular the associations of urinary hippurate with measures of alpha diversity [34,51,52].

With formate and 4-hydroxyphenylacetate, we identified two novel urinary markers of microbial alpha diversity. Formate is a known microbial fermentation product of a wide range of microbes across various phyla [53,54] (Table S8). However, formate is also a product of human metabolism, e.g., as a by-product of multiple human pathways, such as tryptophan degradation, sterol metabolism, and one-carbon metabolism [53]. Thus, while it is plausible that urinary formate is a marker of microbial diversity, the relatively small effect size indicates that most of the variance in urinary formate concentration was due to host factors. Yet, urinary formate may be an interesting candidate for further research into host–microbiome metabolic interactions.

4-Hydroxyphenylacetate is an intermediate of tyrosine metabolism, both in humans and in microbes. However, the production of 4-hydroxyphenylacetate is a rather rare capacity among the microbes in AGORA2 (333 strains noted; in comparison, 6010 strains can produce formate and 1552 can produce benzoate; for complete lists, see Supplementary Table S8). Moreover, several important opportunistic pathogens are among the 4-hydroxyphenylacetate-producing species, such as *Burkholderia cepacia* [55], *Acinetobacter baumannii* [56], *Clostridioides difficile* [57], and *Pseudomonas aeruginosa* [58]. Notably, the association between microbiome diversity and urinary 4-hydroxyphenylacetate was negative, allowing for the speculation that 4-hydroxyphenylacetate is a metabolic marker of dysbiosis, in particular given the prominence of pathogens in the list of microbes capable of producing 4-hydroxyphenylacetate. Nevertheless, urinary 4-hydroxyphenylacetate may be largely determined by host factors, as we failed to predict a substantial amount of variance in urinary 4-hydroxyphenylacetate levels using genus abundances. However, it should be noted that small intestinal bacterial overgrowth in children has been associated with a marked increase in 4-hydroxyphenylacetate in urine [59].

A further result was the association between *Escherichia/Shigella* and indoxyl-3-sulfate, although sensitivity analyses indicated that this specific result needs careful interpretation. *Escherichia/Shigella* is a Gram-negative opportunistic pathogen [60], which is implicated in a variety of intraabdominal infections and is linked to gut microbiome instability [33]. It is also an important producer of pro-inflammatory lipopolysaccharides [61]. Indoxyl-3-sulfate is a metabolite produced by the liver through sulfation of the microbial fermentation product indole, which is produced during tryptophan degradation by various microbes, including members of the genera *Escherichia* and *Shigella*. Thus, while not reported previously, the association is plausible given the known metabolic traits of the associated genera. Importantly, indoxyl-3-sulfate is a known uremic toxin that accumulates in chronic kidney disease and is also said to be pro-inflammatory in general [62]. Thus, our results corroborated the role of *Escherichia/Shigella*, one of the most important opportunistic pathogens in the gut microbiome, in the production of indoxyl-3-sulfate with potential adverse clinical implications.

The main results of our study, however, were with respect to the metabolite hippurate. This metabolite was found to have a strong positive association with microbial alpha diversity, as well as a rich association pattern with various genera. These results provide an additional validation of earlier studies [34,51,52]. Thus, cumulative evidence points towards hippurate being a marker of alpha diversity and gut microbiome metabolism. Moreover, hippurate was also shown to be associated with metabolic health [63,64], and certain experimental evidence exists showing beneficial effects in mice models of diabetes [52]. Hippurate is produced via glycine conjugation of benzoate (Figure 3B), predominantly in the liver, although the pathway was also reported for the kidney [65]. Benzoate can either be directly ingested through the consumption of benzoate-containing food, such as berries, seafood, and dairy products, or produced by the microbiome by metabolising other phenols [66]. However, benzoate is also degraded by microbes in various pathways, including pathways that feed benzoate into central carbon metabolism [61]. Notably, the pathways for benzoate production and benzoate degradation co-occur in microbes [38]. Previous studies have shown that the gut microbiome has a clear effect on human benzoate pools [61]. Thus, the question arises whether the microbiome is a net consumer or producer of benzoate. In this respect, the analyses of the metagenomic data from Yachida et al. [39], which we functionally annotated using the AGORA2 resource [38], pointed towards the gut microbiome being a net consumer of benzoate. As our analyses demonstrated, highly diverse communities had lower reaction abundances of benzoate-transporting reactions. Thus, it follows plausibly that the higher the diversity, the lower the capacity to metabolise benzoate. Less benzoate consumption by the gut microbiome could then result in more benzoate being transported through the portal vein to the liver for hippurate formation. An important observation in this context is that benzoate exhibits antimicrobial activity such that high benzoate diets may lead to microbiomes with high-benzoate-metabolising capacities [66]. Although this hypothesis needs further validation, it would have consequences for interventional studies aiming at promoting human health through the microbiome. Our analyses would indicate that reducing microbial benzoate metabolism could be beneficial through the effects of hippurate.

Finally, our results indicated that the microbiome-related metabolic traits associated with urinary hippurate were of interest for human health, as they were robustly associated with markers of inflammation, liver injury, and metabolic health. As such, they fully mediated the effects of microbial diversity on the same markers, indicating that microbial benzoate metabolism is one of the metabolic functions underlying the rich associations of microbial diversity to human health.

Although our study successfully replicated known findings while expanding the scope of knowledge regarding metabolome–microbiome relations, certain strengths and limitations have to be kept in mind. First, while our study was of reasonable size, the statistical power of finding associations was low for rare genera and metabolites with missing quantifications. Moreover, since the human microbiome shows strong regional differences, generalisations of our results towards other regions are not guaranteed, and an independent validation investigating the herein-reported biomarkers is needed [67,68]. Moreover, due to the usage of 16S rRNA gene sequencing, the taxonomic resolution was limited to the genus level in most cases. Therefore, it was not possible to investigate species or strain-specific microbe–metabolite associations. Importantly, our analyses were cross-sectional in design, and while we could adjust for a wide range of covariates relevant to metabolomics and microbiome data, there was still the risk of unmeasured confounding. Moreover, our analyses of metabolite–microbiome relations were performed on a predominantly healthy population, which limits the ability to detect associations with manifest diseases. As our study was a fasting study, certain aspects of diet–microbiome-host interactions could not be investigated. In relation to microbial benzoate metabolism, it can be conceived that our current knowledge is still incomplete [61]. Thus, the hypothesis regarding the relationship between benzoate metabolism and alpha diversity based on functional annotations representing our current knowledge base needs further validation, preferably in an experimental set-up.

In conclusion, our study demonstrated the usefulness of NMR urine metabolomics in the assessment of host–microbiome interactions in the domain of metabolism. As NMR is inherently robust, reproducible, non-destructive, and involves minimal sample preparation, we believe NMR metabolomics will have a future in the emerging field of microbiome–host interaction in health and disease.

## 4. Materials and Methods

### 4.1. Study Population

Data of SHIP-TREND (recruited 2008–2012, *n* = 4420) was used, consisting of individuals from the region of Pomerania, North-Eastern Germany. The SHIP cohorts were designed to investigate the prevalence and incidence of clinical and subclinical phenotypes and their risk factors. For detailed information on the design, biomaterials, and available data, see [35].

In the presented study, we focused on the sub-cohort of SHIP-TREND with available 16S rRNA gene-sequencing data of stool samples (*n* = 3637) and the sub-cohort with NMR urine metabolomics (*n* = 996). Both 16S rRNA gene sequencing data and NMR metabolomics were available for 951 individuals. Due to the design of the SHIP-TREND study, individuals with known diabetes were not characterised using NMR metabolomics. This was the only exclusion criteria. However, as a consequence, the SHIP-TREND NMR sample was healthier than the average population, as diabetes-related comorbidities and traits were underrepresented.

The institutional review board of the University of Greifswald approved the survey and methods of the SHIP studies and all analyses followed the Declaration of Helsinki. Written informed consent was provided by all participants.

### 4.2. Assays and Phenotypes

The medical history and sociodemographic factors of each participant were evaluated using a computer-assisted face-to-face interview. Next, participants underwent extensive medical examinations, including measurements of the waist circumference, body height weight, and blood pressure. Waist circumference was used as an indicator of abdominal fat in all analyses. Participants were asked to bring their medication prescription sheets or packing containers of all medication they have been taken within the last seven days. By counting the number of alcoholic beverages that had been consumed on average per day over the last 30 days, alcohol consumption was estimated.

Blood samples were drawn between 07:00 a.m. and 12:30 p.m. Fasting time was assessed by asking for the last time the participants ate or drank beverages other than water. In SHIP-TREND, participants were explicitly asked not to eat or drink before blood sampling. Fasting time was an average of 13:28 h, standard deviation (SD) = 1:32 h. Blood samples were taken from the cubital vein and analysed directly or stored at −80 °C in the Integrated Research Biobank of the University Medicine Greifswald [69]. Spontaneous urine specimens were collected and immediately stored at −80 °C. White blood cell count (WBC), red blood cell count (RBC), and thrombocytes count (PLT) were measured on the XE 5000 from Sysmex (Sysmex Deutschland GmbH, Norderstedt, Germany). Glycated hemoglobin (HbA1c) concentrations were determined using high-performance liquid chromatography (Bio-Rad Diamat, Munich, Germany). Highly sensitive CRP, triglycerides, total cholesterol (TC), high-density lipoprotein cholesterol (HDL-C), low-density lipoprotein cholesterol (LDL-C), GGT, ASAT, ALAT, and creatinine (Jaffé) were determined on the Dimension VISTA 1500 according to the recommendations of the manufacturer (Siemens Healthcare Diagnostics GmbH, Eschborn, Germany). From the serum creatinine, the estimated glomerular filtration rate (eGFR) was calculated using the Modification of Diet in Renal Disease equation, as described before [35]. All requirements of the corresponding guideline according to quality specifications were at least fulfilled. Plasma fibrinogen concentrations were assayed according to Clauss (BCS, Siemens Healthcare Diagnostics GmbH; Eschborn). Exocrine pancreatic function was determined using a pancreatic elastase ELISA (BIOSERV Diagnostics, Greifswald, Germany) based on faecal samples, as described before.

### 4.3. 16S rRNA Gene Sequencing and Taxonomic Assignments

Stool sample sequencing followed established procedures, as outlined before in detail [70]. Briefly, study participants collected faecal samples at home and stored them in a tube containing a stabilising DNA buffer. Next, faecal samples were transported to our laboratory. Then, DNA from the faecal samples was extracted (PSP Spin Stool DNA Kit; Stratec Biomedical AG, Birkenfeld, Germany) and stored at −20 °C until analysis using 16S rRNA gene sequencing of the V1–V2 region utilising a MiSeq platform (Illumina, San Diego, CA, USA). For taxonomic assignments, DADA2 16 (V.1.10) was employed for amplicon data processing, enabling single-nucleotide resolutions of amplicons. All samples were normalised to 10,000 16S rRNA gene read counts.

### 4.4. NMR Measurements in SHIP-TREND

After thawing, urine specimens were centrifuged for 5 min at 3000× *g*, and the supernatant was used for spectroscopic analysis. To this purpose, we mixed 450 µL of urine with 50 µL of phosphate buffer to stabilise the urinary pH at 7.0 (±0.35). The buffer was prepared with D_2_O and contained sodium 3-trimethylsilyl-(2,2,3,3-D4)-1-propionate (TSP) as a reference. Spectra were recorded on a Bruker DRX-400 NMR spectrometer (Bruker BioSpin GmbH, Ettlingen, Germany) at a ^1^H frequency of 400.13 MHz. The instrument was equipped with a 4 mm selective inverse flow probe (FISEI, 120 µL active volume) with a z-gradient. Specimens were automatically delivered to the spectrometer via flow injection. The acquisition temperature was set to 300 K. A standard one-dimensional ^1^H-NMR pulse sequence with suppression of the water peak (NOESYPREAST) was used: RD − P(90°) − 4 µsec − P(90°) − tm − P(90°) − acquisition of the free induction decay (FID). The non-selective 90° hard pulse P(90°) was adjusted to 9.4 µsec. The relaxation delay (RD), mixing time (tm), and acquisition time were set to 4 s, 100 msec, and 3.96 s, respectively, resulting in a total recycle time of ~8.0 s. Low-power continuous-wave irradiation on the water resonance at a field strength of ~25 Hz was applied during RD and tm for pre-saturation. After the application of 4 dummy scans, 64 FIDs were collected into 65,536 (64 K) complex data points using a spectral width of 20.689 parts per million (ppm). FIDs were multiplied with an exponential function corresponding to a line broadening of 0.3 Hz before Fourier transformation. Spectra were processed within TOP-SPIN 1.3 (Bruker BioSpin, Billerica, MA, USA).

The NMR spectra were analysed by Chenomx Inc. (Edmonton, AB, Canada) and manually annotated by spectral pattern matching using the Chenomx Worksuite 7.0 (Chenomx Inc., Edmonton, Canada) to deduce absolute urinary metabolite concentrations. The method of Chenomx’s patented NMR-based metabolomics platform is based on software and an extensive reference database that contains a compound list of over 300 metabolite models and was used for the analysis. Manual phasing, baseline correction, and targeted metabolic profiling using the Chenomx library were performed. This resulted in a list of 60 identified metabolites (including creatinine, which was used for normalisation) and their concentrations in millimoles per liter (mM). The spectrum regions of water (δ = 4.6–5.0) and the regions below δ = 0.0 and above δ = 10.0 were removed from the analysis for all groups in order to prevent variation in each sample. Each NMR variable was normalised to the total area in order to allow for a spectrum-to-spectrum comparison.

### 4.5. Data Normalisation and Outlier Detection

For statistical analyses, regression-based normalisation was performed on the log-transformed urinary concentrations based on probabilistic quotient normalisation using restricted cubic splines, as described in [36]. Regression-based normalisation leads to slightly higher statistical power, and it accounts for potential metabolite-specific dilution–concentration dependencies [36]. Details on regression-based normalisation can be found in Hertel et al. [36]. After the regression-based normalisation, outliers based on the 4-standard-deviation rule were excluded.

### 4.6. Statistical Analyses in SHIP-TREND

For descriptive statistics, metric variables were described using means and standard deviations, while categorical and ordinal variables were described using proportions. All reported *p*-values were two-tailed, and multiple testing correction was performed via applying the false discovery rate (FDR). All major routes of analysis were performed through multivariable regressions from the class of generalised linear models. All regression analyses included a basic set of covariates if not specified otherwise, consisting of age, sex, age–sex interaction terms, and waist circumference. Furthermore, we included smoking, hypertonia, HbA1c, years of education, the eGFR, urinary pH, and alcohol intake as covariates for the full adjustment. Age, waist circumference, and eGFR were introduced as restricted cubic splines using four knots at the 5% percentile, the 33% percentile, the 66% percentile, and the 95% percentile of the respective distributions. Nonlinear modelling for these variables was chosen, as previous analyses indicated nonlinearity in respect to basic covariates [71].

In the first set of regression models, we screened urinary metabolite concentrations on associations with genus abundances. We included only metabolites and genera with at least 50% non-zero measurements, resulting in 1681 metabolite–genus combinations tested. This rather strict criterion was applied to filter out metabolite–genus combinations with low case numbers and thus low statistical power. The regressions then included normalised metabolite concentration as a response variable, while the genus abundance was utilised as a predictor of interest adjusting for the set of basic covariates. Lastly, we used the 16S rRNA gene-sequencing batch variable as a random effect variable to account for batch effects. The herein identified metabolite–genus pairs with FDR < 0.05 were then subject to sensitivity analyses using the full list of covariates. Furthermore, we utilised non-parametric bootstrapping (2000 replications) for recalculating *p*-values, ensuring that our results are not biased by certain distributional features of the microbial abundances. In the main text, we report the fully adjusted effects, whereas Supplementary Tables S2 and S3 contain the summary statistics for all regressions performed.

In the second set of analogous regression models, we exchanged the genus abundance with a binary variable indicating genus presence or absence. Here, we included all genera that were found in at 20% of the samples and maximally in 80% of the samples. This led to 1848 genus–metabolite pairs. As before, these combinations were screened on significant metabolite–genus associations using the basic set of covariates, and significant associations were then re-tested using the full adjustment.

Third, to test the associations between the urine metabolome and metrics of gut microbiome alpha diversity, we ran mixed-effect regression models as before. However, now the predictor of interest was either the Shannon entropy or the species richness, as calculated via the Vegan R package [72]. Multivariable linear mixed regressions were performed for the 42 metabolites having more than 50% non-zero measurements testing on associations between metabolites and alpha diversity measures, both for the basic adjustment and the full list of covariates. After graphical examination of the results indicating the presence of nonlinear diversity, we furthermore checked on nonlinear associations via restricted cubic splines as before.

Fourth, we explored the predictive value of the microbiome for each of the 42 metabolites that was measured in more than 50% of the cases. To this end, we used an elastic net regression methodology, which is a machine learning approach from the class of penalised regressions based on the objective function

(1) $$Q=\frac{1}{2n}\overset{n}{\underset{i=1}{\sum }}{\left(y_{i}-\beta_{0}-x_{i}\beta^{\prime }\right)}^{2}+\lambda \overset{p}{\underset{j=1}{\sum }}\left(\frac{1-\alpha }{2}\beta_{j}{}_{\;}^{2}+\alpha |\beta_{j}|\right)$$

where *n* is the sample size, *y* is the response variable (the normalised metabolite concentration), *x* is the vector of predictors (genus abundances), *p* is the number of predictors, *β* is the regression coefficients, and *λ* and *α* are the penalty parameters. Then, a grid search over various *λ* and *α* values was performed using internal cross-validation to find the parameters with the lowest prediction error. Note that if *α* = 1, the elastic net is equivalent to the Lasso, while with *α* = 0, it becomes equivalent to the Ridge regression. We performed a grid search on *α* = {0, 0.1, …, 1} and using the default parameters for *λ* in the STATA 17/mp elasticnet function. We utilised 10-fold cross-validation and utilised the out-of-sample R-squared value as an indicator of model fit. Full results for all 42 metabolites can be found in Supplementary Table S6.

As only the microbiome-based prediction score for urinary hippurate explained the substantial proportions of variance, we explored the information encoded in this prediction score further. To this end, we utilised linear mixed-effect regressions with either the normalised urinary hippurate concentrations, the Shannon entropy, or the microbiome-based hippurate prediction score as a response variable, the full set of covariates, and one additional clinical parameter as a predictor of interest. In this way, we screened inflammation markers (high sensitivity CRP, fibrinogen, white blood cell counts), markers of metabolic health (triglycerides, ratio of total cholesterol and HDL cholesterol, HbA1c), and markers of liver injury (GGT, ASAT, ALAT) on the association with urinary hippurate concentration, the Shannon entropy, and the microbiome-based hippurate prediction score. Finally, we performed mediation analyses following the procedure of Hicks et al. [73] to test whether the microbiome-based hippurate prediction score mediates the effects of microbiome diversity on markers of human health.

### 4.7. Statistical Analyses on Yachida et al.’s Metagenome Data

To enlighten the relation between microbial benzoate metabolism and microbial alpha diversity further, we went on to analyse a public metagenomics dataset (*n* = 616, see Yachida et al. [39] for details) with functional annotations based on the AGORA2 platform [40]. In brief, species abundances were retrieved via the MetaPhlaN2 pipeline and then mapped onto AGORA2. AGORA2 is a collection of semi-automatically derived and manually curated genome-scale reconstructions of microbes that was curated against known experimental data reflecting the true metabolic capacities of the microbes [40]. We utilised the genome-scale reconstructions to analyse which microbes were able to secrete benzoate, formate, and 4-hydroxyphenylacetate. Moreover, we calculated reaction abundances for all reactions involving benzoate based on the AGORA2 functional annotations using established pipelines [74] for all metagenomes measured in Yachida et al. [39]. Then, we regressed the reaction abundances via fractional regressions on the Shannon entropy including age, sex, body mass index, and the case–control status as covariates.

## Figures and Tables

**Table 1:** Descriptive statistics for the utilised SHIP-TREND-0 sub-cohorts.

	SHIP-TREND with Faecal Samples (*n* = 3637)		SHIP-TREND with Faecal Samples and NMR Metabolite Measurements (*n* = 951)	
Variable	Missing Values, %	Mean (SD) or Share, %	Missing Values, %	Mean (SD) or Share, %
Age, years	0.00	51.33 (14.94)	0.00	50.21 (13.63)
Female, %	0.00	51.69%	0.00	56.68%
Body mass index, kg/m^2^	0.16	28.02 (5.15)	0.00	27.37 (4.57)
Waist circumference, cm	0.27	90.79 (14.35)	0.00	88.08 (12.82)
Current smoking, %	0.25	26.82%	0.11	21.79%
Average alcohol consumption over the last 30 d, g/d	0.91	8.83 (13.79)	0.63	8.56 (13.31)
Diabetes ^a^	0.16	11.54%	0.00	2.73%
Hypertonia ^b^	0.33	46.43%	0.11	39.58%
HbA1c, %	0.19	5.34 (0.83)	0.11	5.19 (0.56)
eGFR, mL/min	0.16	89.73 (18.81)	0.00	92.12 (17.12)
White blood cell count, Gpt/L	1.95	6.08 (2.70)	0.21	5.73 (1.48)
Triglycerides, mmol/L	0.16	1.64 (1.24)	0.00	1.42 (0.86)
Ratio of TC/HDL-C	0.16	4.03 (1.26)	0.00	3.93 (1.14)
Fibrinogen, g/L	2.64	3.07 (0.74)	0.95	3.02 (0.73)
CRP (high sensitive), mg/L	4.67	2.52 (3.93)	3.36	2.29 (3.67)
GGT, μkat/L	0.19	0.70 (0.80)	0.00	0.65 (0.63)
ALAT, μkat/L	0.22	0.45 (0.30)	0.11	0.44 (0.29)
ASAT, μkat/L	0.30	0.33 (0.19)	0.21	0.32 (0.17)

^a^ Diabetes is defined by either an HbA1c > 6.5% or intake of antidiabetic medication. ^b^ Hypertonia is defined by the intake of antihypertensive medication or blood pressure higher than 140/90 mmHg; SHIP—Study of Health in Pomerania, SD—standard deviation, CRP—C-reactive protein, TC—total cholesterol, HDL-C—high-density lipoprotein cholesterol, HbA1c—glycated hemoglobin, GGT—gamma-glutamyl-transferase, ALAT—alanine-amino-transferase, ASAT—aspartate-amino-transferase.

**Table 2:** Biomarker associations from multivariable regressions for urinary hippurate, Shannon entropy, and the microbiome-based hippurate prediction score.

	Urinary Hippurate (*n* = 951)		Shannon Entropy (*n* = 3637) ^b^		Microbiome-Based Hippurate Prediction Score (*n* = 3637) ^b^	
Marker	b (95% CI) *	*p*-Value *	b (95% CI) *	*p*-Value *	b (95% CI) *	*p*-Value *
Log hs-CRP	**−0.09 (−0.17, −0.01)**	**2.40 × 10^−2^**	−0.01 (−0.03, 0.00)	7.55 × 10^−2^	**−0.01 (−0.02, −0.00)**	**1.65 × 10^−2^**
Fibrinogen	−0.02 (−0.09, 0.04)	4.59 × 10^−1^	−0.00 (−0.02, 0.01)	6.27 × 10^−1^	0.01 (−0.00, 0.02)	2.05 × 10^−1^
White blood cell count	−0.15 (−0.28, −0.03)	1.68 × 10^−2^	−0.00 (−0.01, 0.00)	1.76 × 10^−1^	−0.00 (−0.01, 0.00)	3.76 × 10^−1^
Triglycerides	−0.02 (−0.09, 0.05)	5.24 × 10^−1^	**−0.03 (−0.04, −0.02)**	**3.83 × 10^−6^**	**−0.03 (−0.04, −0.02)**	**3.35 × 10^14^**
Ratio of TC/HDL-C	0.04 (−0.05, 0.12)	3.95 × 10^−1^	−0.01 (−0.02, 0.00)	1.58 × 10^−1^	−0.00 (−0.01, 0.01)	8.08 × 10^−1^
Baseline glucose	−0.03 (−0.09, 0.02)	1.87 × 10^−1^	0.00 (−0.01, 0.01)	9.32 × 10^−1^	−0.00 (−0.01, 0.00)	1.67 × 10^−1^
HbA1c	0.004 (−0.04, 0.05)	8.45 × 10^−1^	0.01 (−0.01, 0.03)	2.13 × 10^−1^	0.01 (−0.00, 0.02)	1.69 × 10^−1^
Log GGT	**−0.05 (−0.09, −0.01)**	**2.05 × 10^−2^**	**−0.07 (−0.10, 0.04)**	**1.64 × 10^−7^**	**−0.10 (−0.12, 0.08)**	**4.13 × 10^22^**
Log ALAT	−0.03 (−0.06, 0.01)	1.07 × 10^−1^	**−0.04 (−0.07, 0.01)**	**9.85 × 10^−3^**	**−0.07 (−0.09, 0.04)**	**4.77 × 10^−9^**
Log ASAT	−0.03 (−0.06, 0.00)	6.29 × 10^−2^	−0.02 (−0.06, 0.01)	1.62 × 10^−1^	**−0.06 (−0.08, 0.03)**	**4.33 × 10^−6^**

* Estimates from multivariable (mixed-effect ^b^) linear regressions including age (nonlinear), sex, age–sex interaction terms, waist circumference (nonlinear), smoking, hypertonia, years of education, kidney function (nonlinear), urinary pH, and alcohol intake. b—unstandardised regression coefficient, 95% CI—95% confidence interval, hs-CRP—high-sensitivity C-reactive protein, TC—total cholesterol, HDL-C—high-density lipoprotein cholesterol, HbA1c—glycated hemoglobin, GGT—gamma-glutamyl transferase, ALAT—alanine aminotransferase, ASAT—aspartate aminotransferase. Bold indicates statistical significance.

## Data Availability

The data presented in this study are not publicly available due to the data use policy of SHIP. All SHIP data can be requested free of charge from the SHIP transfer office (https://www.fvcm.med.uni-greifswald.de/cm_antrag/index.php accessed on 29 March 2022).

## Supplementary Materials

The following supporting information can be downloaded at: https://www.mdpi.com/article/10.3390/metabo12040308/s1, Table S1: Descriptive statistics on urinary metabolites that were included and excluded from the analyses; Table S2: Association analyses between genus presences and urinary metabolite concentrations; Table S3: Association analyses between genus presences and urinary metabolite concentrations; Table S4: Summary statistics for metabolite Shannon diversity associations; Table S5: Summary statistics for metabolite species richness associations; Table S6: Overview on performances on predicting metabolite concentrations from genus abundances via elastic net regression methodologies; Table S7: Sample characteristics of the utilised metagenomic data from Yachida et al. (2019); Table S8: Lists of strains from AGORA2 producing benzoate, formate, and 4-hydroxyphenylacetate.
